# WaSP-ECG: A Wave Segmentation Pretraining Toolkit for Electrocardiogram Analysis

**DOI:** 10.3389/fphys.2022.760000

**Published:** 2022-03-17

**Authors:** Rob Brisk, Raymond R. Bond, Dewar Finlay, James A. D. McLaughlin, Alicja J. Piadlo, David J. McEneaney

**Affiliations:** ^1^Faculty of Computing, Engineering and the Built Environment, Ulster University, Belfast, United Kingdom; ^2^Cardiology Department, Craigavon Area Hospital, Craigavon, United Kingdom

**Keywords:** artificial intelligence, electrocardiogram (ECG), machine learning, explainable AI, representation learning

## Abstract

**Introduction:**

Representation learning allows artificial intelligence (AI) models to learn useful features from large, unlabelled datasets. This can reduce the need for labelled data across a range of downstream tasks. It was hypothesised that wave segmentation would be a useful form of electrocardiogram (ECG) representation learning. In addition to reducing labelled data requirements, segmentation masks may provide a mechanism for explainable AI. This study details the development and evaluation of a Wave Segmentation Pretraining (WaSP) application.

**Materials and Methods:**

*Pretraining:* A non-AI-based ECG signal and image simulator was developed to generate ECGs and wave segmentation masks. U-Net models were trained to segment waves from synthetic ECGs. *Dataset:* The raw sample files from the PTB-XL dataset were downloaded. Each ECG was also plotted into an image. *Fine-tuning and evaluation:* A hold-out approach was used with a 60:20:20 training/validation/test set split. The encoder portions of the U-Net models were fine-tuned to classify PTB-XL ECGs for two tasks: sinus rhythm (SR) vs atrial fibrillation (AF), and myocardial infarction (MI) vs normal ECGs. The fine-tuning was repeated without pretraining. Results were compared. *Explainable AI:* an example pipeline combining AI-derived segmentation masks and a rule-based AF detector was developed and evaluated.

**Results:**

WaSP consistently improved model performance on downstream tasks for both ECG signals and images. The difference between non-pretrained models and models pretrained for wave segmentation was particularly marked for ECG image analysis. A selection of segmentation masks are shown. An AF detection algorithm comprising both AI and rule-based components performed less well than end-to-end AI models but its outputs are proposed to be highly explainable. An example output is shown.

**Conclusion:**

WaSP using synthetic data and labels allows AI models to learn useful features for downstream ECG analysis with real-world data. Segmentation masks provide an intermediate output that may facilitate confidence calibration in the context of end-to-end AI. It is possible to combine AI-derived segmentation masks and rule-based diagnostic classifiers for explainable ECG analysis.

## Introduction

### Artificial Intelligence for Electrocardiography

#### Overview

Correct electrocardiogram (ECG) interpretation is key to the diagnosis and treatment of myocardial infarction (MI) and life-threatening arrhythmias, among many other conditions (Macfarlane et al., 2010). Computerised ECG analysers have been in existence for over 50 years (Rautaharju, 2016). However, semantic interpretation of ECG data requires the identification of subtle patterns from a complex signal. It is challenging to describe this process in conventional computer code.

Artificial intelligence (AI) can perform strongly in this field because it does not rely on the ability of human experts to expound process knowledge. AI-enabled analysis has led to state-of-the-art performance across a range of ECG interpretations tasks (Kashou et al., 2020).

#### Types of Artificial Intelligence for Electrocardiogram Interpretation

Machine learning (ML)-based AI refers to a set of automated statistical modelling techniques. AI models learn through trial and error. At each step of the learning process, the model makes a prediction. An error is calculated based on a loss function. A new set of model parameters is discerned using an optimisation function. Further steps are taken until some endpoint is reached (Svensén and Bishop, 2007).

Deep learning (DL) is the frontier of modern AI. DL arose from the study of artificial neural networks (ANNs) (Goodfellow et al., 2016). ANNs are computational graphs comprising densely interconnected multi-layer perceptrons (MLPs). They are inspired by the biological brain.

The difference between “classical” ML and DL is often summarised thus: ML techniques generally rely on prior processing of input data to extract key features using expert domain knowledge; DL techniques learn end-to-end processing, which includes feature extraction (Rusk, 2016). In practice, this results in a trade-off: DL techniques are able to detect more complex patterns in higher dimensional data compared with ML approaches, and can function with lower signal-to-noise ratios (SNRs), but at the cost of being less explainable.

In the domain of ECG processing, it is the feature extraction step that presents the greatest challenge for conventional (non-AI) applications. Filtering noise and other electrical artefact from ECG signals, then identifying key features such as the primary waves, has been a major research theme in automated ECG analysis for decades (Luo and Johnston, 2010) but is by no means a solved problem. This limits the utility of ML algorithms (Ribeiro et al., 2020), where knowledge-based feature extraction remains an important part of the pipeline. It is here that DL algorithms can excel.

Convolutional neural networks (CNNs) are a variant of ANNs, and a form of DL. They leverage large numbers of learnable convolutional filters to detect important signals within high-noise data (Goodfellow et al., 2016). They were developed primarily for semantic analysis of real-world images (Krizhevsky et al., 2012), but the technology transfers well to ECG signals and has been applied to a broad range of clinical problems (Lopez-Jimenez et al., 2020; Makimoto et al., 2020; Siontis et al., 2021). This includes a landmark 2019 study by Hannun et al. (2019) that claimed “cardiologist level” diagnosis of atrial fibrillation (AF), and even a study later that year by Attia et al. (2019) that described a DL algorithm able to detect incipient AF. It is likely that convolutional filters in the earlier layers of a CNN learn filtering methods to deal with common ECG noise and artefact, such as baseline wander, powerline interference and non-cardiac muscle activity. This reduces or negates the need for traditional filtering methods (Arsene et al., 2019).

Transformer neural networks emerged from the field of natural language processing (NLP). They use attention mechanisms to parallelise sequential data processing. Attention mechanisms can evaluate the relative importance of distant features within data, whereas CNNs have a limited capacity for this. This can be advantageous when relationships between non-local features are important, such as in multi-clauses sentences or even entire documents (Vaswani et al., 2017). However, it has recently been shown that transformer models can scale to sizes up to hundred of billions of trainable parameters with a relatively linear improvement in performance (Korngiebel and Mooney, 2021). The sheer power of these “mega-AI models” means that they are beginning to attain state-of-the-art performance in domains where CNNs have traditionally dominated, such as image processing (Dosovitskiy et al., 2020). Transformers for ECG signal analysis is an active research area (Yan et al., 2019; Natarajan et al., 2020), and it may be that this is the place to look for the next wave of breakthroughs in this field.

### Challenges

#### Data Paucity

As AI models grow larger and more sophisticated, they need more data to maximise their learning potential. This challenge is being actively addressed by the creation of large public datasets such as PhysioNet’s PTB-XL (Wagner et al., 2020). However, large transformer models can require billions of training samples to reach their full potential, which is orders of magnitude beyond the largest public databases at present. In rarer ECG conditions, data paucity remains a bottleneck to training even small AI models. It is also a challenge for ECG image analysis, where SNR is much lower than in raw signal format, and where more training data are needed to compensate for this (Brisk et al., 2019).

#### Explainable Artificial Intelligence

Elucidating the process logic encoded by networks comprising millions of parameters is extremely difficult. This is often referred to as the “black box effect,” and has given rise of a field of study known as explainable AI (Samek et al., 2019). The black box effect can make it difficult for humans to exercise oversight of an AI system’s decision logic. Without this oversight, it is challenging to calibrate one’s confidence in the outputs of an AI systems. Confidence calibration is known to play a key role in ECG interpretation (Bond et al., 2018).

### Related Work

#### Overcoming Data Paucity

Representation learning (RL) lessens the need for labelled training data. In RL, an AI model is trained for a task that forces it to learn useful “latent representations” of the data without manually assigned labels. This can be hard to intuit for non-data scientists, and a full explanation is beyond the scope of this article. Interested readers are directed to an excellent review by Bengio et al. (2013). RL is used for some of the most sophisticated AI models in existence today (Vaswani et al., 2017). Models pre-trained using RL and then fine-tuned for specific tasks using labelled training data, which is to say that they undergo a further training period for a specific task with constraints placed upon the rate at which they learn (Komodakis and Gidaris, 2018). The constrained learning rate means that the fine-tuning period serves to refine the latent representations acquired during pretraining, rather than simply overwriting previous representations with new ones. This latter phenomenon is known as “catastrophic forgetting” (Kirkpatrick et al., 2017).

Representation learning has been investigated in the domain of ECG interpretation by a small number of studies. A recent example is from Sarkar and Etemad (2020). They tasked a model with identifying which augmentations had been applied to ECG signals, such as addition of Gaussian noise or signal flipping. This reduced the need for labelled data when fine-tuning for downstream tasks. However, this is a sparsely explored topic to date.

### Explainable Electrocardiogram Artificial Intelligence

Several approaches to explainable AI-enabled ECG analysis have been investigated. A recent paper by Maweu et al. (2021) infers the relative importance of key ECG waves with respect to an AI model’s output (Dosovitskiy et al., 2020). This approach of retrospectively interrogating trained models to infer logic processes is widely used. Our group has previously explored one such technique known as saliency mapping. It was found that the outputs provided false reassurance, in that they appeared to show that the AI model was leveraging the ST segment to diagnose acute myocardial ischaemia. This supported the idea that the model was leveraging features in the input data known to relate closely to the target label, whereas is was later discovered that this was not the case (Maweu et al., 2021).

A study by Jo et al. (2021) prioritises explainable outputs at the algorithm design stage. They detect AF by using two linked AI models. One is for detecting the presence or absence of P waves. The other is for detecting regular or irregular R–R intervals. This follows the established decision logic of clinical experts and results in relatively interpretable outputs. It is unclear that this approach would generalise well to more complex diagnostic patterns.

### Focus of This Work

Wave identification is a fundamental step for any ECG analysis by a human expert. Therefore, it was hypothesised that an AI model pretrained to segment key waves from ECG signals will:

1. Learn useful representations of ECG data and train more efficiently for downstream tasks, minimising the need for manually labelled data.
2. Provide a human-readable intermediate output that may facilitate confidence calibration.
3. Facilitate a choice between using DL technology as a feature extractor for explainable downstream analysis, or using DL for end-to-end ECG analysis by fine-tuning the pretrained models.

The challenge was that manual segmentation of waves within 12-lead ECGs is very laborious. This is particularly true of ECG images, which the authors have previously proposed as an important modality that has been under-represented within ECG AI research to date, and which was to be used in this study along with raw sample data. RL approaches generally leverage self- or semi-supervised methods to make pretraining on large datasets practical, and manual wave segmentation was not felt to be practical for this experiment.

This led to a further hypothesis, whose evaluation is proposed as the most significant contribution of this study to the field:

1. Representations learned from pretraining on synthetic data and labels will transfer to downstream tasks using real ECG data.

## Materials and Methods

### Overview

The following approach was designed to test the hypotheses described above. Steps 2–7 were repeated for raw sample and image formats. Steps 8 and 9 were only undertaken for the image-based experiment.

1. Develop an ECG and segmentation mask generator.
2. Train an AI model to predict segmentation masks using a synthetised dataset: referred to hereafter as Wave Segmentation Pretraining or WaSP.
3. Predict segmentation masks for a database of real ECGs (for analysis at step 7).
4. Fine-tune the model for downstream diagnostic tasks using database of labelled real ECGs.
5. Re-initialise the model with pre-WaSP weights and train this model for downstream diagnostic tasks using database of labelled real ECGs.
6. Compare the results from steps 4 and 5 to test hypothesis (1).
7. Undertake a qualitative analysis of segmentation masks from step 3 to test hypothesis (2).
8. Develop and evaluate a rule-based diagnostic pipeline to evaluate hypothesis (3).
9. Train a “mixed modality” model for diagnostic tasks, whereby an ECG signal is read back from the predicted segmentation mask and fed into a 1D AI model.

### Terminology

A segmentation mask is a set of labels that overlays some input data, denoting the semantic category to which each datum belongs. In the case of image data, the segmentation mask has the same height and width as the pixel array of the original image. Where the original image contains colour channel values at each position in the pixel array, however, the segmentation mask contains an integer value. This value denotes the semantic class to which each pixel belongs. In the case of a single-class segmentation task – for example, segmenting human faces from photographs – all pixels belonging to a face will be represented by a 1, whereas all other pixels will be considered as background and will be assigned a 0. For the purposes of this experiment, the following target classes were defined:

0 = background

1 = P wave

2 = P-R interval

3 = QRS complex

4 = ST segment

5 = T wave

6 = T-P segment

7 = T/P overlap

Downstream tasks can be any task for which a pretrained AI model is subsequently re-trained. In the case of this experiment, these tasks are described in section “Fine-Tuning for Diagnostic Classification.”

### Synthetic Electrocardiogram Generation

Rob Brisk developed an application to simulate 12-lead ECG signals. The Python programming language was used. The aim of the simulator development was to produce a broad spectrum of realistic ECG phenotypes. The parameters determining rhythm and morphology of ECGs were governed by pseudo-random number generation to ensure each ECG was unique. Random noise and baseline wander were added to each signal. Voltages were scaled randomly.

In effect, the simulator was a form of expert system (Jackson, 1986) informed by key works in the field such as Macfarlane et al. (2010), in addition to the author’s own experience as a practising cardiologist. The ECG signals were optionally plotted into 12-lead ECG images. Segmentation masks were generated for each ECG signal and image.

### Wave Segmentation Pretraining

#### Model Architecture

**FIGURE 1:**
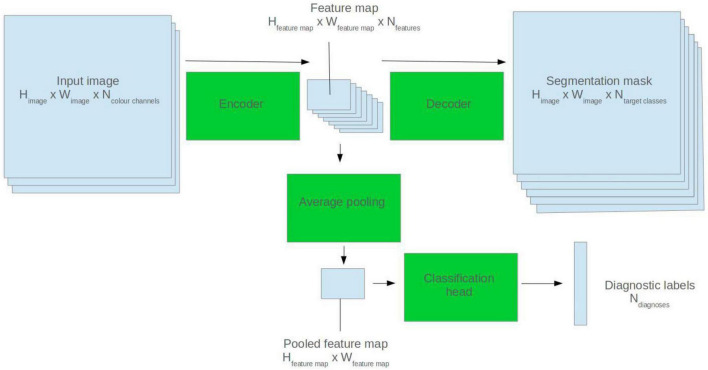
Illustration of how a 2D U-Net model can be applied to segmentation and classification tasks.

U-Net model architectures were used for ECG segmentation. The U-Net is a popular CNN-based architecture for image segmentation. It comprises two halves: an encoder and a decoder. The encoder abstracts high level features from the input image. The decoder generates a segmentation mask based on the encoder feature map (Ronneberger et al., 2015). See Figure 1 for a visual depiction.

The encoder used for each model was based on the SEResNet architecture (Hu et al., 2018). This is one of many permutations of the “vanilla” CNN. A full review of CNN types is beyond the scope of this work, though such reviews exist (Khan et al., 2020). SEResNet was felt to represent a demonstrably performant architecture that would fit with the compute constraints of the experiment. The signal-based model used a 1D U-Net with a SEResNet encoder. The image-based model used a 2D U-Net with a SEResNet152 encoder.

The 1D models were initialised with random parameter values (commonly known as model weights). The 2D models were initialised with weights derived from real-world image classification training with the ImageNet database (Deng et al., 2009).

#### Training Protocol

For the self-supervised pretraining, 32,000 ECGs and segmentation masks were synthesised. The segmentation models were trained during a single pass through the dataset (known as an epoch). A dice loss function was use with Jaccard smoothing (Bertels et al., 2019). Hyperparameters (parameters that control the training process, rather than forming part of the model itself) were manually tuned based on the training loss, training F1 score (see Equation 1) and a visual inspection of segmentation masks at the end of each training cycle.


(1) $$\frac{2\times Sensitivity\times PPV}{Sensitivity\times PPV}$$

F1 score (PPV, positive predictive value).

An enhanced pretraining step was undertaken as an additional experiment. A further 12,000 ECGs and segmentation masks were synthesised. Each ECG showed either sinus rhythm (SR) or AF. Each ECG also showed one of six morphological phenotypes: normal, left anterior hemiblock, left posterior hemiblock, high take-off, left bundle branch block, or anterior ST-elevation. A classification head was added to the model encoder to predict the rhythm and morphological phenotype of each ECG. The model was simultaneously trained for both segmentation and classification using a multi-task learning approach.

### Fine-Tuning for Diagnostic Classification

The PhysioNet PTB-XL database was downloaded, along with the label files (Wagner et al., 2020). This is one of the largest publicly available repositories of labelled ECG signals, comprising 21,837 ECGs from 18,885 subjects. The labels for each ECG include one or more of 71 ECG-SCP statements, and each ECG is assigned one of five diagnostic superclasses. The raw samples were converted to NumPy arrays. They were also plotted into ECG images using the software developed for this experiment.

Two diagnostic classification tasks were undertaken: SR vs AF and normal morphology vs MI. For each of these tasks, the signals were divided into training, validation and test sets using a 60:20:20 split. A hold-out test set approach was used.

To fine-tune the models for diagnostic classification, average pooling was applied to the output of the final convolutional filter of the U-Net encoder. Two densely connected layers were appended, and a sigmoid activation function applied to the output nodes. See Figure 1 for a visual representation.

### Rule-Based Atrial Fibrillation Detector

**FIGURE 2:**
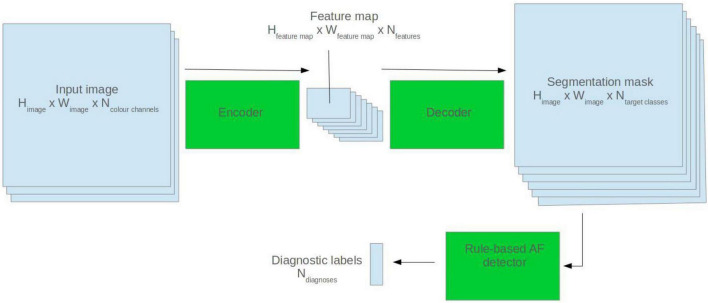
Visualisation of the rule-based AF detector.

To create the rule-based AF detector, segmentation masks were predicted for ECG images using the pretrained 2D U-Net model. A rule-based algorithm was used to determine the locations of QRS complexes, based on clusters of pixels assigned to the QRS class. The standard deviation of the R–R intervals was calculated. The area approximately 250 mS prior to each QRS complex was evaluated for the presence of a P wave, based on cluster of pixels assigned to the P wave class. See Figure 2 for a visualisation.

If the number of QRS complexes preceded by a P wave was less than threshold X, and the standard deviation of R–R intervals was greater than threshold Y, the ECG was classified as AF. Thresholds X and Y were set using a brute force search on the validation set, where the combination maximising the F1 score was selected.

### Mixed Modality Model

**FIGURE 3:**
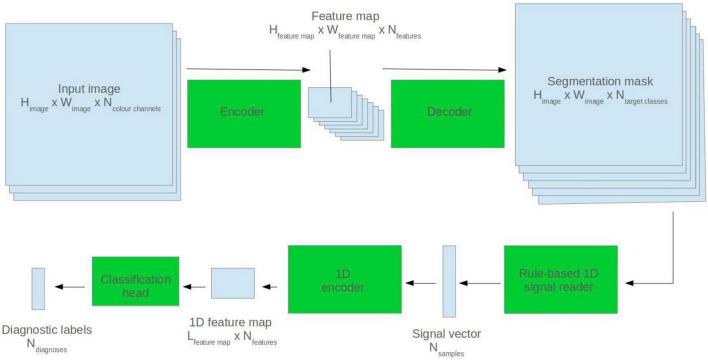
Visualisation of the mixed modality analyser.

Segmentation masks were predicted for ECG images using the pretrained 2D U-Net model. A rule-based algorithm was used to read back the ECG signal. This employed a grid search method described by this group in a previous paper (Brisk et al., 2019). The extrapolated ECG signal was fed into a 1D ResNet encoder which made a diagnostic prediction. See Figure 3 for a visualisation.

### Analysis

Sensitivity, specificity, positive predictive value and F1 score were calculated with respect to the AF and MI classes. The F1 score was used as the primary metric for comparing the models and testing hypothesis (1). Training loss curves were plotted. No additional statistical analysis was undertaken.

This experiment resulted in three sets of results for each of the two diagnostic tasks for the raw samples dataset:

1. Results from the non-pretrained model.
2. Results from the model pretrained using wave segmentation.
3. Results from enhanced pretraining.

For the ECG image dataset, three additional sets of results were produced:

1. Results from a model initiated with random weights (as opposed to ImageNet weights) without WaSP.
2. Results from the mixed modality model.
3. Results from the rule-based AF detector.

Segmentation masks for selected ECGs from the PTB-XL dataset were predicted at the end of pretraining. Another set of masks were predicted after fine-tuning the models. The segmentation masks predicted by the raw samples model were transposed into images for manual inspection.

A small subset of ECG images were printed and either photographed or scanned. Segmentation masks were predicted using the pretrained 2D U-Net model to evaluate robustness to real-world image artefact.

## Results

### Data

#### Electrocardiogram Generator

The source code for the ECG generator can be found here: https://github.com/docbrisky/WaSP-ECG

#### Synthetic Dataset

**FIGURE 4:**
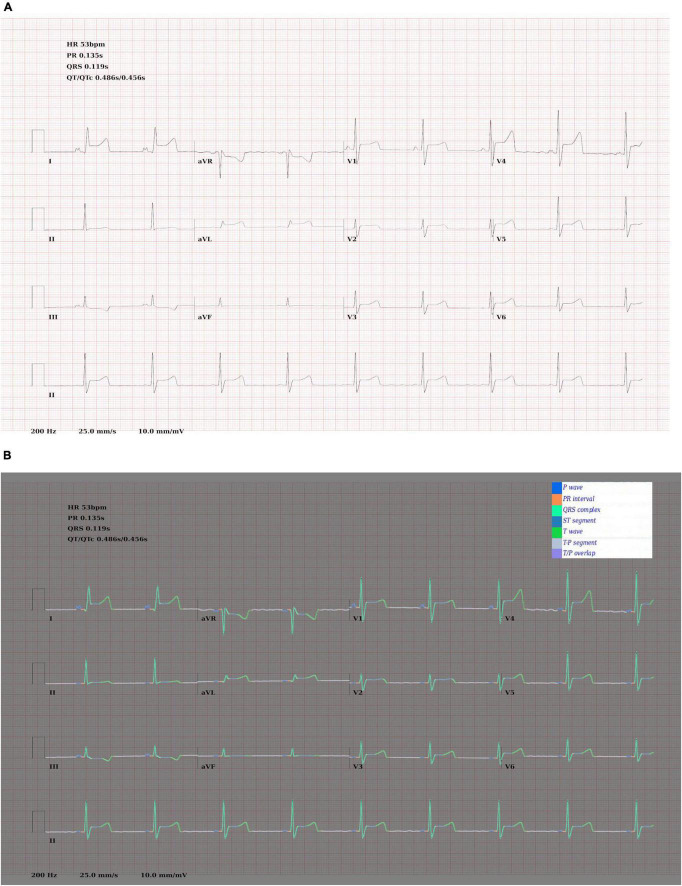
**(A)** Synthetic ECG showing SR with anterior ST elevation. **(B)** The same image with the ground truth wave segmentation mask superimposed.

Examples of ECGs and segmentation masks produced by the ECG generator can be seen in Figure 4.

#### Real Dataset

Characteristics of the PTB-XL database are described by Wagner et al. (2020).

### Segmentation Pretraining

**FIGURE 5:**
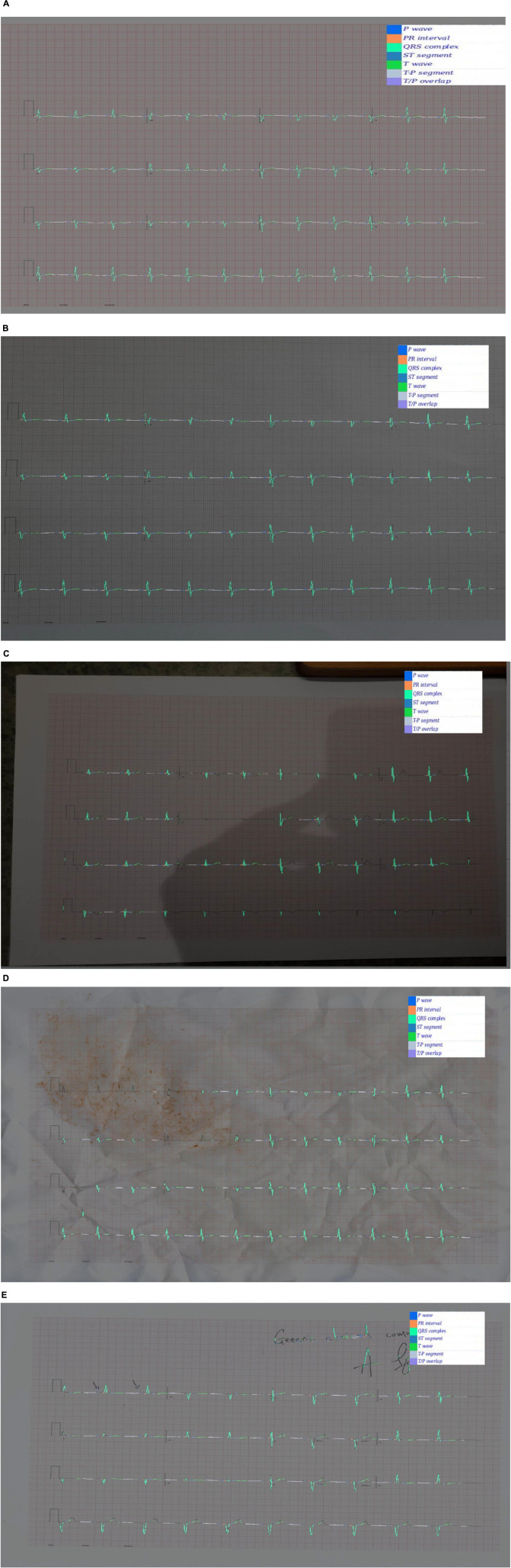
**(A)** A segmentation mask for a randomly selected ECG signal from the PTB database. This mask was predicted for the raw ECG signal using a 1D U-Net. Both the raw signal and the segmentation mask were subsequently plotted into an image file. The model that predicted this mask was pretrained exclusively on synthetic ECG signals. **(B)** Segmentation mask for a randomly selected PTB signal that was (i) plotted into an ECG image using the software developed for this experiment; (ii) printed using a standard desktop printer (HP Envy 4520 series); photographed using a Samsung Galaxy S10 mobile phone (flash off, bright daylight). The mask was then predicted by a 2D U-Net model that had been pretrained exclusively on synthetic data. **(C)** This segmentation was produced using the same process as **(B)**, except that the photograph was taken in more challenging lighting conditions (at night, flash off, xenon strip lighting with shadows on image). **(D)** This segmentation was produced using the same process as **(B)**, except that the printed ECG was (i) crumpled up; (ii) sprinkled with coffee; (iii) smeared with tomato sauce; (iv) scanned using an HP Envy 4520 desktop scanner (at 600 DPI). This process was the result of a discussion about how to recreate a level of image artefact that might represent real-world clinical practice. David J. McEneaney noted that he is regularly asked to review ECGs that have been stained with blood or coffee, and occasionally ECGs that have been thrown in the bin and subsequently retrieved. **(E)** This segmentation was produced using the same process as **(B)**, except that manual annotation artefact was added and the image was scanned using an HP Envy 4520 series scanned (at 600 DPI).

Examples of predicted segmentation masks for real ECGs can be seen in Figures 5A–E. This includes predicted segmentation masks for ECG images that were printed and either photographed or scanned, some with additional artefact added. The models pretrained exclusively on synthetic data were felt to generalise well to real-world ECG data. Robustness to image artefact was variable.

### Fine Tuning

**FIGURE 6:**
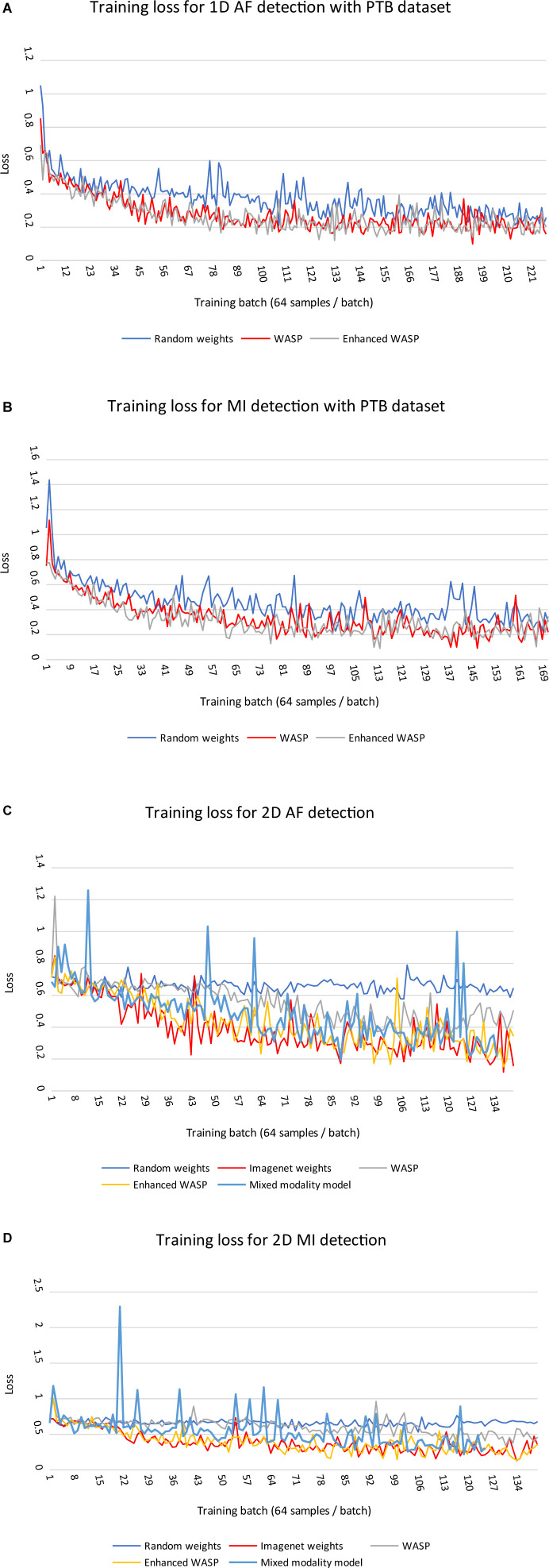
**(A,B)** Training losses for 1D models on diagnostic classification tasks with PTB ECGs. Each training run comprised a single epoch. WASP, WaSP. **(C,D)** Training losses for 2D models on diagnostic classification tasks with PTB ECGs.

Loss curves for 1D and 2D models can be seen in Figure 6A–D. Across modalities, models with randomly initialised weights converged more slowly than pretrained models. Among the 2D models, those with weights derived from non-enhanced WaSP converged more slowly than either models that had undergone enhanced pretraining or models that were initialised with ImageNet-derived weights.

### Diagnostic Classification

The sensitivity, specificity, positive predictive value, and F1 scores for the diagnostic tasks can be seen in Table 1.

**TABLE 1:** Test set results for the two diagnostic classification tasks.

**MI detection 1D**	**Random weights**	**WASP**	**Enhanced WASP**			
Sensitivity	0.906077	0.955801	0.909761			
Specificity	0.840292	0.83142	0.914927			
PPV	0.762791	0.762675	0.858384			
F1	0.828283	0.848386	0.883326			

**AF detection 1D**	**Random weights**	**WASP**	**Enhanced WASP**			

Sensitivity	0.658537	0.764228	0.853659			
Specificity	0.989232	0.996874	0.991316			
PPV	0.723214	0.912621	0.807692			
F1	0.689362	0.831858	0.83004			

**MI detection 2D**	**Random weights**	**Imagenet weights**	**WASP**	**Enhanced WASP**	**Mixed modality model**	

Sensitivity	0	0.824125	0.513812	0.848066	0.667587	
Specificity	1	0.950939	0.964509	0.935282	0.950418	
PPV	0	0.904954	0.891374	0.88134	0.884146	
F1	0	0.862651	0.651869	0.864383	0.760756	

**AF detection 2D**	**Random weights**	**Imagenet weights**	**WASP**	**Enhanced WASP**	**Mixed modality model**	**Rule-based model**

Sensitivity	0	0.798611	0.395833	0.801105	0.657459	0.53125
Specificity	1	0.987541	0.991991	0.947808	0.968163	0.957876
PPV	0	0.845588	0.808511	0.896907	0.92129	0.518644
F1	0	0.821429	0.531469	0.846304	0.767329	0.524871

*Highest and lowest F1 scores for each set of results are highlighted in green and yellow, respectively.*

For the ECG images, the models that underwent enhanced pretraining achieved the highest F1 score in both AF and MI detection. For the raw samples, the enhanced pretrained model scored highest for MI detection. The unenhanced pretrained model scored highest for AF detection.

For the ECG image set, the non-pretrained models predicted all samples as normal. Consequently, the sensitivity and positive predictive value for both models was zero. The rule-based AF detector scored lower than any pretrained model, although the F1 scores for the rule-based detector and the unenhanced pretrained model were close at 0.52 and 0.53, respectively. The mixed modality model outperformed the rule-based and unenhanced pretrained models, but underperformed the enhanced pretrained and ImageNet-trained models.

### Confidence Calibration and Explainable Outputs

**FIGURE 7:**
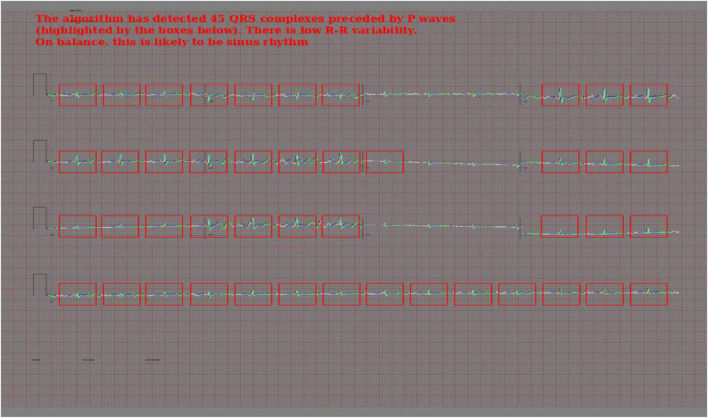
Output of the rule-based AF detection algorithm. The authors propose that this is highly explainable compared with end-to-end AI analysis.

**FIGURE 8:**
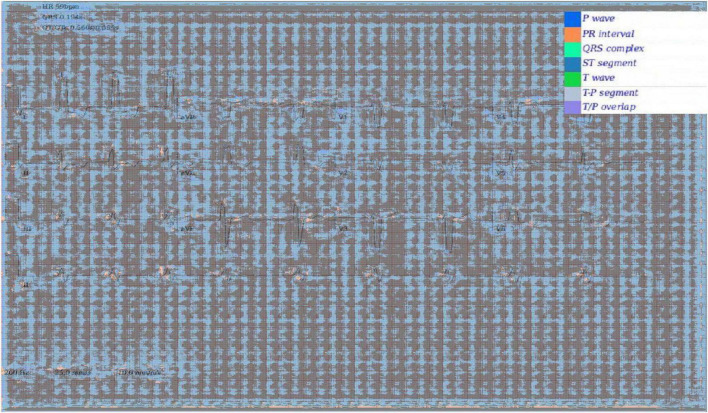
Segmentation mask produced by a 2D model initiated with ImageNet weights, but not having undergone any further training on ECG segmentation nor classification tasks.

In addition to Figures 5A–E, which show examples of segmentation masks, Figure 7 shows an example output from the rule-based AF classifier. Figure 8 shows a segmentation mask produced by a model that has been newly initialised with ImageNet weights. This model can be assumed to have no diagnostic capabilities with respect to ECG analysis. It is proposed that this segmentation mask would cause a clinician to place low confidence in the model’s outputs, whereas the segmentation masks shown in Figures 5A,B may warrant relative high confidence. The segmentation masks in Figures 5C–E may alert the clinician to some issues caused by image artefact, and trigger additional caution when considering the model’s final diagnostic output.

## Discussion

### Key Conclusions

This study shows that WaSP using a synthetic dataset can improve training efficiency for downstream ECG tasks with real ECG data. The impact of pretraining was particularly marked with ECG image analysis. WaSP also enables meaningful intermediate output from the AI model.

The rule-based AF detection algorithm demonstrated a novel approach to ECG image analysis that benefits from advances in modern AI but is proposed to be highly explainable. Accuracy was limited but refinement of the technique may result in performance improvements.

Reading back signals from ECG image segmentation masks allowed a 1D classifier to detect both MI and AF with moderate accuracy. This shows that the SNR within the extrapolated data is high enough to facilitate some degree of downstream analysis. The motivation for investigating this is discussed in section “Future Work.”

### Limitations

The diagnostic tasks chosen for this study are not representative of the spectrum of clinical ECG phenotypes encountered in real-world practice. Two relatively easy diagnostic tasks were chosen to minimise confounding factors and facilitate a head-to-head comparison of different pretraining approaches. The absolute results from these tasks add little to the field; rather, it is intended that the relative results serve as an early evaluation of WaSP and of pretraining with synthetic ECG data. More work is needed to determine whether the findings of this study would generalise to a wider range of diagnostic problems.

One of the stated motivations for investigating WaSP was that it may facilitate clinician confidence calibration. The figures shown in this study may enable readers to begin forming their own conclusions on this matter. However, this hypothesis was not formally evaluated and can be considered unproven to date.

This study was undertaken in a retrospective observational setting. A single dataset was used for training, testing and validation. There is an increased risk of over-fitting a particular data distribution in this context. Results shown here may not generalise to other datasets or populations.

For the diagnostic classification evaluation, ECG images were plotted directly from the signals *in silico*. In a clinical setting, ECG images would be printed and either scanned or photographed. This would introduce image artefact that may alter the accuracy of downstream tasks, as illustrated in Figure 5. For any future work aiming to establish whether the novel image-based techniques described here are useful for downstream clinical applications, it is likely that the full evaluation would need to be conducted with paper ECGs.

The rule-based AF detector was not evaluated with the 1D signals as this would have required a substantial re-write of the application, which was not felt to be warranted as there are already many rule-based AF detection algorithms for raw sample data.

### Comparison With Existing Approaches

As discussed during the “Introduction” section, approaches to both pretraining and explainable DL for ECG analysis have been explored by other groups. To the best of our knowledge, however, this is the first demonstration that pretraining with synthetic data is effective.

This has potentially significant implications for the fast-growing field of ECG AI. The increasing number of large public ECG databases like PTB-XL is helping to drive research in this field. However, such databases are finite and may be subject to bias: centres with the expertise and resources to produce such datasets tend to exist in more affluent global regions and may over-represent certain demographic groups; rare diseases and paediatric conditions are often under-represented in such biobanks (reference); studies from patients suffering with emerging diseases that may have cardiac involvement (e.g., COVID-19) may take some time to reach these datasets.

Knowledge-based engineering of synthetic datasets allows much greater control over the distribution of covariates-of-interest within the training data. This can help to counterbalance bias and to increase the occurrence of rare but important features. It can also facilitate the creation of much larger datasets than would be possible using real patient data. Historically, supplementing labelled datasets with synthetic samples for task-specific training has been problematic (Sankaranarayanan et al., 2018). For learning general representations during pretraining, however, we propose that a lower fidelity is acceptable: the model will learn additional or altered features that occur in real-world datasets during fine-tuning.

### Additional Points of Interest

Electrocardiogram image models pretrained on ImageNet performed significantly better than models initialised with random weights. This implies that some features learned from analysing photographs of real-world scenes transfer well to ECG analysis.

Performance worsened when the ImageNet-trained models underwent non-enhanced WaSP. A possible explanation for this is that WaSP caused catastrophic forgetting. It may be possible to overcome this issue by freezing early convolutional layers and reducing the learning rate (Shmelkov et al., 2017).

Enhanced WaSP involved the addition of a diagnostic labelling task in addition to wave segmentation; the model was asked to output both types of label for each sample using an approach known as “multi-task learning.” This seemed to improve performance significantly compared with non-enhanced WaSP. The same black box nature of AI that was one of the motivating factors for this study makes it difficult to ascertain exactly why this was the case. However, the authors posit that the addition of a diagnostic label for the whole ECG forced the model to learn about relationship between more distant parts of the ECG (for example, the diagnosis of left bundle branch block requires that the model evaluate the QRS-T morphology in multiple leads simultaneously), whereas wave segmentation can be achieved by leveraging only very local parts of the data.

### Relevance of This Work to the Wider Field

As state-of-the-art AI models grow in size and complexity, more training data is required to capitalise on their increased pattern recognition capabilities (Li et al., 2019). In this study, WaSP expedited convergence during fine-tuning and produced higher results after a single training epoch. Therefore, WaSP can reduce the need for labelled training to produce equivalent results. This approach may allow larger AI model architectures to be used for ECG tasks where there would otherwise be insufficient labelled training data.

Explainable AI is an active research topic in healthcare (Amann et al., 2020). Mechanisms by which clinicians can calibrate confidence or review decision logic may provide key to adoption of AI in practice. The work undertaken for this study may catalyse future research into segmentation masks as a mechanism for confidence calibration in ECG analysis, and mixed AI and rule-based analysis as a mechanism for explainable ECG image analysis.

The code base for this experiment has been published under a permissive open source licence. The application has been named WaSP-ECG. The intention is to facilitate reproduction of results and accelerate future research in the field. The inclusion of Zero optimisation functionality in the code base (Rajbhandari et al., 2020) allows researchers to train larger models on their existing infrastructure than would have otherwise been possible, or to use higher resolution input data. This may allow researchers to extend existing AI techniques and improve model performance.

### Future Work

Only two rhythm types and six morphological phenotypes were simulated during the enhanced pretraining phase of this study. Given the performance improvement observed with enhanced pretraining over non-enhanced WaSP in the context of ECG images, it may be that a wider repertoire of simulated ECG phenotypes would further improve downstream performance.

The robustness of AI techniques to image artefact (see Figure 5) was felt by the authors to be limited. The ability to photograph ECG images on a mobile phone and upload for cloud-based analysis is proposed to be a worthwhile goal, as it would decrease the dependence on hardware-bound analysers. This, in turn, would allow for more agile development of novel ECG applications and easier integration with multi-model clinical data, such as symptomatology, biochemical results, cardiac imaging, etc. There is an emerging body of evidence that fusing multi-modal data leads to improved performance of medical AI systems (Huang et al., 2020). For this reason, investigating approaches to improve robustness to image artefact, challenging lighting conditions, etc., may be a valuable research avenue.

Evaluating WaSP for diagnostic tasks more representative of real-world clinical problems would be a key next step for the line of investigation presented in this study. The use of data from additional patient populations and evaluation of diagnostic capabilities in a prospective setting would help to establish the generalisability of the results presented here.

## Data Availability Statement

Publicly available datasets were analysed in this study. This data can be found here: https://physionet.org/content/ptb-xl/1.0.1/.

## Author Contributions

RBr designed and executed the experiment and wrote the first draft of the manuscript. RBo reviewed and advised on the design of the experiment, and provided technical input during the execution of the experiment. DF, JM, AP, and DM reviewed and advised on the design of the experiment and the write-up of the manuscript. All authors contributed to the article and approved the submitted version.

## Conflict of Interest

The authors declare that the research was conducted in the absence of any commercial or financial relationships that could be construed as a potential conflict of interest.

## Publisher’s Note

All claims expressed in this article are solely those of the authors and do not necessarily represent those of their affiliated organizations, or those of the publisher, the editors and the reviewers. Any product that may be evaluated in this article, or claim that may be made by its manufacturer, is not guaranteed or endorsed by the publisher.
